# Would they do it again? Final treatment decisions in malignant brain tumour patients—a caregiver’s perspective

**DOI:** 10.1007/s00520-022-06796-y

**Published:** 2022-01-21

**Authors:** Marion Rapp, Christiane von Sass, Clara Backhaus, Daniel Hänggi, Marcel Alexander Kamp, Michael Sabel

**Affiliations:** 1 Centre for Neuro-Oncology, Heinrich-Heine-University Clinic, Moorenstr. 5, 40225 Duesseldorf, Germany; 2 Department of Neurosurgery, Heinrich-Heine-University Clinic, Moorenstr. 5, 40225 Duesseldorf, Germany; 3 Department of Neurosurgery, University of Jena, Jena, Germany

**Keywords:** Malignant brain tumour, End-of-life phase, Caregiver, Neurooncology, Treatment decisions

## Abstract

**Purpose:**

Overall survival of malignant brain tumour patients has significantly been increased over the last years. However, therapy remains palliative, and side effects should be balanced. Once terminal phase is entered, both patients and caregivers may find it hard to accept, and further therapies are demanded. But little is known about this highly sensitive period. Therefore, we analysed the last therapy decisions from the family caregiver’s perspective. Would they support their beloved ones in the same way or would they now recommend a different therapy decision?

**Methods:**

Caregivers of deceased malignant brain tumour patients, treated at our neurooncological centre between 2011 and 2017, were included. We designed a questionnaire to analyse the impact of the last therapy decision (resection, chemotherapy, radiotherapy), focusing on probable repeat of the choice taken and general therapy satisfaction. Independent variables, for example “satisfaction with therapy”, were analysed using linear regression analysis, the coefficient of determination *R*^2^ and the standardized regression coefficient *β*. The binary logistic regression analyses were taken to illustrate relationships with the dichotomously scaled outcome parameter “re-choice of therapy”. Odds ratio analyses were used to determine the strength of a relationship between two characteristics.

**Results:**

Data from 102 caregivers (life partners (70.6%)) were analysed retrospectively. Each 40% of patients died in a hospice or at home (20% in a hospital). In 67.6% the last therapy was chemotherapy followed by radiotherapy (16.7%) and surgery (15.7%). A positive evaluation of the last therapy was significantly correlated to re-choosing of respective therapy (chemo-/radiotherapy: *p* = 0.000) and satisfaction with informed consent (*p* = 0.000). Satisfaction regarding interpersonal contact was significantly correlated to satisfaction with resection (*p* = 0.000) and chemotherapy (*p* = 0.000 27 caregivers (28.7%) felt overburdened with this situation).

**Conclusion:**

This analysis demonstrates a significant correlation between a positive relation of patient/caregiver/physician and the subjective perception of the latest therapy. It underlines the central role of caregivers, who should be involved in therapy discussions. Neurooncologists should be specially trained in communication and psycho-oncology.

**Supplementary Information:**

The online version contains supplementary material available at 10.1007/s00520-022-06796-y.

## Introduction

Due to improved and aggressive treatment options, overall survival of malignant glioma patients is significantly increased from 14.6 to 48.1 months [1–4] during the last decade—however, therapy still remains palliative. Therefore, therapy should be balanced between increasing lifetime and decreasing quality of life. Tumour recurrence with its destructive growth pattern is inevitable, and patients will develop neurologic symptoms as well as neurocognitive impairment during the course of disease. Over time, initial tumour-specific treatment, which is mainly guided by special neurooncologists will transform into best supportive-care treatment, which is usually conducted by general practitioners or palliative healthcare personnel [5], finally leading over to the end-of-life (EOL) phase. The EOL phase is defined as time prior to death when symptom load increases and antitumoural therapy is no longer effective. It may range from days to weeks but is generally entered within 3 months from death [6]. There is no clear definition when the EOL phase of oncologic patients begins; it commonly depends on neurological deficits, the patients’ general state of health and his will [7]. Most of the time, the EOL phase treatment is determined by palliative and/or supportive care. But in some cases, patients and their caregivers are explicitly seeking for further treatment options mainly because they are not willing to stop active treatment phase [8, 9].

Therefore, this crucial phase is also challenging for neurooncologists, who have to balance therapeutic side effects, quality of life and the hope of eventually increasing overall survival. In this vulnerable phase, physicians also have to deal with demanding patients and their caregivers who maybe deny accepting the EOL. Little is known about how this phase affects neurooncologic patients and their caregivers. Data mainly concentrate on additional patient-centred care and caregivers’ specific burdens [10–12].

Here, we were interested in the caregivers’ retrospective evaluation of the final tumour-specific treatment before entering the EOL phase. Would the caregivers support the same therapy decisions, with the knowledge of both side effects and possible extension of life time? Or would they now tend to stop active treatment earlier? Therefore, we performed a caregiver survey, whose relative died from a progressive malignant brain tumour and was treated at our neurooncological centre. The self-designed questionnaire mainly focused on the subjective experience of the final tumour-specific treatment. So, would they do it again?

## Patients and methods

### Ethics approval

The study was approved by the local ethics committee (study number 5338, March 2016). All caregivers obtained and gave informed consent. Withdrawal was possible at any time on the caregivers’ request.

### Data source

We conducted a cross-sectional survey study of caregivers with the following inclusion criteria: caregivers of (1) recurrent malignant brain tumour patients, (2) who were offered a neurooncological treatment at the neurooncological centre of the university hospital of Duesseldorf and (3) died due to recurrent brain tumour between 2011 and 2017.

### Study design

Caregivers were contacted via telephone prior to receive the survey questionnaire. Out of respect, caregivers whose relatives died less than 6 months ago were only contacted via mail. All other caregivers, whose beloved ones passed away > 6 months, were contacted initially via phone to inform them about our survey. Caregivers were asked to complete and return a questionnaire designed as a 20-item assessment plus free text options. The survey was developed in close collaboration with neurosurgeons and neuro- and psycho-oncologists solely for this study and was not validated before (see supplemented material). Questions of the survey included 6 different categories regarding sociodemographic data, satisfaction during the last oncologic therapy, decision of the last oncologic therapy, place of death, overall evaluation and concluding remarks. Answers were designed either as dichotomic (yes/no) or as a 5-point scale (1 = very satisfied; 5 = very dissatisfied). In further analyses, the 5-point scale was merged as follows: “completely content” and “content” were summarized into content and “not content” and “not content at all” into not content.

Additionally, comments could be made using free text. Because of few and heterogenous comments, free text was not included into further analyses.

Patient data regarding histopathological diagnosis, neurological status, age and gender were obtained from the clinical database (including MRI scans and medical reports from outpatient examination as well as inpatient examinations).

All data were correlated and analysed with focus on the last neurooncologic therapy, defined as the final active treatment related to the brain tumour, which was performed by the neurooncological department. Following this therapy, no other palliative treatment was provided.

### Statistical analysis

All statistical analyses were carried out using IBM Statistics, version 24 (SPSS IBM Corp., Armonk, NY, USA). A *p* value of 0.05 was set as statistically significant. Due to a high number of tested hypotheses, the problem of multiple testing was corrected with the Bonferroni correction. Corrected *P* level was set at 0.0025. Data was described by standard statistics, using absolute and relative frequencies for categorical variables and median for continuous variables. Independent variables, such as the (pseudo) metric and Likert-scaled questions about “satisfaction with therapy”, were analysed using linear regression analysis, the coefficient of determination *R*^2^ and the standardized regression coefficient *β*. The binary logistic regression analysis and its outcome the odds ratio (OR) was taken to illustrate relationships with the dichotomously scaled outcome parameter “re-choice of therapy”.

## Results

Between 2011 and 2017, caregivers of 215 neurooncologic patients were asked to take part in the survey. Finally, data of 102 caregivers (47.4%) could be analysed (mainly spouses (*n* = 72, 70.6%)). Caregivers, who were initially contacted via phone, returned the questionnaire more often (*n* = 66, 84.4%), compared to caregivers, who were initially contacted solely by mail (*n* = 36, 26.2%).

**Fig. 1:**
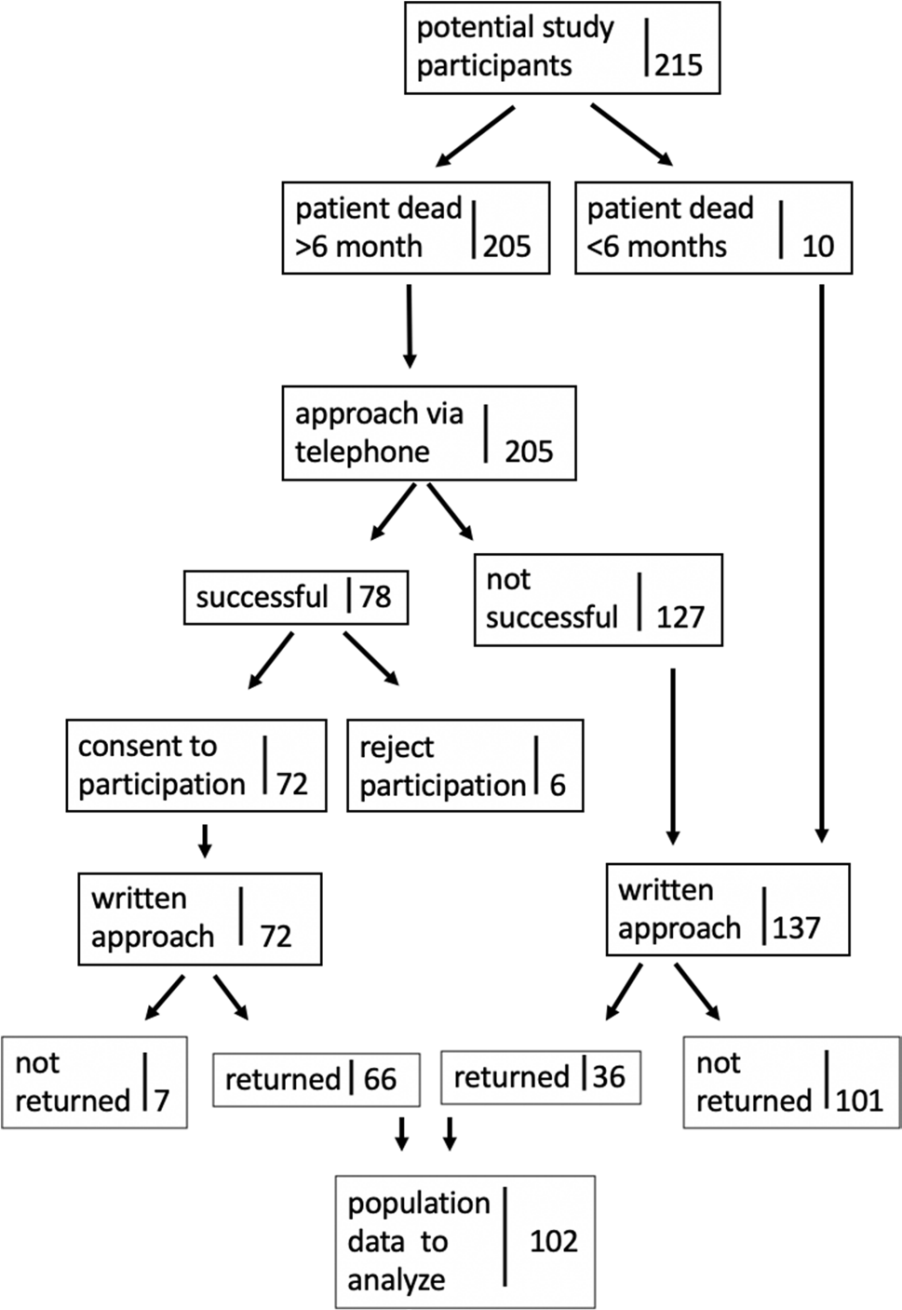
Caregiver recruitment flowchart

Time from assessment to the patient’s death was median 2.8 years (range 6 months–6 years). A caregiver recruitment flowchart is illustrated in Fig. 1.

The mean age of patients was 57.3 years (range 24–79); 69.6% (*n* = 71) were male. The majority of patients was diagnosed with a high-grade glioma (*n* = 85, 83.3%) and 17 patients (16.7%) with cerebral metastasis. The most common final neurooncological therapy was oral chemotherapy (temozolomide) (69 patients (67.6%)), followed by re-radiation (17 patients (16.6%)) and surgical recurrent tumour resection (16 patients (15.6%)). The Karnofsky performance status (KPS) was higher than 70 in 87 patients (85.2%) at the time of therapy initiation. The majority of patients died at home or in a hospice (*n* = 37/*n* = 38, 37.8%/38.8%, respectively); only 23 patients (23.4%) died in a hospital. The majority of caregivers received further care support (*n* = 72, 70.5%).

Detailed clinical and sociodemographic data of patients and caregivers are summarized in Table 1.

**Table 1:** Clinical and sociodemographic characteristics of patients and caregivers

Age in years (patient/caregiver)	
Mean	57.3/57.6
Range	24–79/27–84
Gender (patient)	
Male	71 (69.6%)
Female	31 (30.4%)
Neuropathological diagnosis	
Anaplastic glioma WHO III	20 (19.6%)
Glioblastoma WHO IV	65 (63.7%)
Cerebral metastasis	17 (16.7%)
Number of therapies (range 1–8)	
> 3 therapies	91 (89.2%)
4–5 therapies	9 (8.8%)
< 6 therapies	3 (2.9%)
Relation caregiver	
Spouse	72 (70.6%)
Child	10 (9.8%)
Parent	13 (12.7%)
Sibling	4 (3.9%)
Other	3 (2.9%)
Final oncological therapy	
Resection	16 (15.7%)
Radiation	17 (16.7%)
Chemotherapy	69 (67.6%)
Place of death	
Home	37 (37.8%)
Hospice	38 (38.8%)
Hospital	23 (23.4%)
Additional care support	72 (73.5%)
Household help	6 (8.3%)
Nursing service	33 (45.8%)
Driving service	9 (12.5%)
Psychooncological support	9 (12.5%)
All	15 (20.8%)
KPS before last oncological therapy	
100	17 (16.7%)
> 90	22 (21.6%)
> 80	28 (27.4%)
> 70	20 (19.6%)
< 70	4 (3.9%)
Missing data	11 (10.8%)

### Caregiver’s evaluation of the interaction patient-caregiver-health personnel

The interaction between health personnel and patient/caregiver was positively evaluated in 81.6% (*n* = 80), which was mainly reflected in the evaluation of the communication of the diagnosis and informed consent (up to 85.6%) as well as about upcoming neurooncological therapies (up to 82.5%). Compared to chemotherapy and surgery, caregivers were less satisfied with the informed consent in the (external) department of radiotherapy (64.8%). Almost the half of caregivers (*n* = 53, 55.8%) were satisfied with the handling of their own anxieties, as well as with the handling of the patient’s anxieties (63%). Half of the caregivers were satisfied with the information they have received regarding the referrals to associated institutions like rehabilitation centres (50%), palliative care units (55%) or hospices (50%).

Detailed information about the caregivers’ evaluation regarding communication between patient-physician and caregiver during the last neurooncological therapy are illustrated in Table 2.

**Table 2:** Caregivers’ evaluation of satisfaction regarding different interaction scenarios between patient-physician and caregiver during the last neurooncological therapy. Answers were merged using the 5-point scale questions. Answer possibilities 1: “completely content” and 2: “content” were summarized in the first column as “content”; 3: neutral (second column); 4: “not content” and 5: “not content at all” were summarized in the third column “not content”

General satisfaction regarding	Content (*n*/%)	In part (*n*/%)	Not content (*n*/%)
Interaction caregiver-patient-physician (*n* = 98)	80 (81.6%)	9 (9.2%)	9 (9.2%)
Interaction caregiver-patient-nursing staff (*n* = 96)	63 (65.6%)	18 (18.8%)	15 (15.6%)
Informed consent about diagnosis			
Comprehensibility (*n* = 97)	83 (85.6%)	7 (7.2%)	7 (7.2%)
Time-point (*n* = 95)	71 (74.8%)	13 (13.7%)	11 (11.5%)
General impression (*n* = 97)	71 (73.2%)	11 (11.3%)	15 (15.5%)
Dealing with questions (*n* = 96)	73 (76%)	15 (15.7%)	8 (8.3%)
Informed consent about therapy			
Re-resection (*n* = 97)	80 (82.5%)	9 (9.3%)	8 (8.2%)
Re-radiation (*n* = 91)	59 (64.8%)	18 (19.8%)	14 (15.4%)
Chemotherapy (*n* = 90)	65 (72.2%)	14 (15.6%)	11 (12.2%)
Dealing with anxiety of			
Patient (*n* = 92)	58 (63%)	15 (16.3%)	19 (20.7%)
Caregiver (*n* = 95)	53 (55.8%)	21 (22.1%)	21 (22.1%)
Referral to associated institutions			
Rehabilitation (*n* = 58)	29 (50%)	12 (20.7%)	17 (29.3%)
Palliative care (*n* = 40)	22 (55%)	6 (15%)	12 (30%)
Hospice (*n* = 36)	18 (50%)	4 (11.1%)	14 (38.9%)

The questionnaire further assessed the caregivers’ subjective evaluation regarding different neurooncologic therapies (surgery, radiation and chemotherapy (Table 3)). Almost 50% of the caregivers (*n* = 44) stated that false hopes were raised by therapy initiation. Overburdened by the circumstances caused by the last therapy (e.g. new neurological deficits) were 28 caregivers (29.7%) during this time. For further estimation of the burden caused by the neurooncological therapy, the questionnaire assessed the subjective wish for either intensified therapy or its denial as well as adverse effects. Overall the caregivers were contented with the course of therapy. Most caregivers (*n* = 68, 73.9%) did not wish an intensified or different (*n* = 56, 65.8%) therapy. The majority (57.6–85.7%) would have supported the same choice for a therapy again and stated that therapeutic results outweighed adverse effects (*n* = 49, 57.6%). For further details, see Table 3.

**Table 3:** Caregivers’ evaluation regarding the different neurooncological therapies

	Yes *n*/(%)	No *n*/(%)
False hope (*n* = 93)	44 (47.3%)	49 (52.6%)
Overburdened by situation (*n* = 94)	28 (29.7%)	66 (70.2%)
Deciding again for		
Resection (*n* = 91)	78 (85.7%)	13 (14.2%)
Radiation (*n* = 85)	49 (57.6%)	36 (42.3%)
Chemotherapy (*n* = 87)	60 (68.9%)	27 (31.1%)
Wish for different therapies (*n* = 85)	29 (34.1%)	56 (65.8%)
Therapy effects prevail adverse effects (*n* = 85)	49 (57.6%)	36 (42.3%)
	Denial of (*n* = 93)	Intensifying (*n* = 92)
Resection	4 (4.2%)	9 (9.7%)
Radiation	22 (23.6%)	6 (6.5%)
Chemotherapy	12 (12.9%)	6 (6.5%)
Comb. resection/chemo	2 (2.1%)	1 (1%)
Comb. resection/radio	1 (1%)	1 (1%)
Comb. radio-/chemo	8 (8.6%)	1 (1%)
All	8 (8.6%)	0
None	36 (38.7%)	68 (73.9%)

### Impact factors concerning general satisfaction

Caregivers who were satisfied with the chosen neurooncologic therapy would choose the respective therapy again (surgery: OR = 4.48, *p* = 0.017; chemotherapy: OR = 13.66,* p* = 0.000; radiotherapy: OR = 10.13, *p* = 0.000). The correlation between therapy-specific satisfaction and positively evaluated informed consent was highly significant (surgery: *R*^2^ = 0.35, *ß* = 0.59, *p* = 0.000; chemotherapy: *R*^2^ = 0.24, *ß* = 0.49, *p* = 0.000; radiotherapy: *R*^2^ = 0.46, *ß* = 0.68, *p* = 0.000).

The caregivers’ assessment of handling anxiety was also significantly correlated with the satisfaction with surgery (*R*^2^ = 0.15, *ß* = 0.39, *p* = 0.000). The parameters “overburdened”, “positive effects outweigh adverse therapy effects” and “false hopes” did not significantly affect therapy-specific satisfaction. One significant factor influencing therapy-specific satisfaction was the interpersonal contact in general, including physicians and other medical staff (Table 4).

**Table 4:** Overview of the statistical analysis of the chosen parameters regarding the different neurooncologic treatments (radiotherapy, resection, chemotherapy) using the Bonferroni correction because of multiple testing. Corrected *P* level was set at 0.0025. Linear regression analysis results, the coefficient of determination *R*^2^ and the standardized regression coefficient *β* are added. The binary logistic regression analysis and its outcome the odds ratio (OR) was taken to illustrate relationships with the dichotomously scaled outcome parameter “re-choice of therapy”. Given variable in italic and bold

			Resection				Radiotherapy				Chemotherapy			
			OR		*P*		OR		*P*		OR		*P*	
Choosing therapy again	***Satisfied with resection*** vs. not satisfied		4.48		0.017									
	***Satisfied with radiotherapy*** vs. not satisfied						10.13		**0.000**					
	***Satisfied with chemotherapy*** vs. not satisfied										13.66		**0.000**	
	***Satisfied with informed consent*** vs. not satisfied		2.67		0.151		3.36		**0.001**		5.55		0.021	
	***Overburdened by situation*** vs. not overburdened		0.42		0.163		0.78		0.606		0.88		0.801	
	***False hopes*** vs. no false hopes		0.59		0.406		0.33		0.018		0.41		0.066	
	***Therapy prevails adverse effects*** vs. adverse effects prevail therapy		2.62		0.151		4.15		0.005		3.18		0.220	
	***Satisfied with dealing with anxiety*** vs. not satisfied		3.72		0.042		1.44		0.415		1.81		0.206	
	***Satisfied with interpersonal contact*** vs. not satisfied													
	*a) ****General***		3.21		0.061		1.32		0.573		1.96		0.197	
	*b) ****Physician***		4.25		0.029		1.24		0.710		0.87		0.828	
	*c) ****Medical personnel***		1.89		0.294		1.10		0.840		1.81		0.225	
	***KPS* > *90*** vs. KPS < 90		0.69		0.554		0.91		0.847		0.97		0.946	
			Resection				Radiotherapy				Chemotherapy			
			*R*^2^	*ß*		*P*	*R*^2^	ß		*P*	*R*^2^	*ß*		*P*
Therapy-specific satisfaction		Satisfied with informed consent	0.35	0.59		**0.000**	0.46	0.68		**0.000**	0.24	0.49		**0.000**
		Overburdened by situation	0.03	− 0.18		0.081	0.00	− 0.06		0.635	0.03	− 0.17		0.163
		False hopes	0.01	− 0.11		0.324	0.04	− 0.20		0.088	0.07	− 0.27		0.016
		Therapy prevails adverse effects	0.01	0.12		0.297	0.03	0.17		0.154	0.03	0.18		0.116
		Dealing with anxiety	0.15	0.39		**0.000**	0.13	0.13		0.263	0.03	0.17		0.126
		Satisfied with interpersonal contact												
		a) General	0.15	0.39		**0.000**	0.04	0.19		0.093	0.12	0.34		**0.002**
		b) Physician	0.20	0.45		**0.000**	0.03	0.17		0.144	0.06	0.25		0.025
		c) Medical personnel	0.09	0.30		0.004	0.07	0.26		0.024	0.11	0.33		0.003
		KPS > 90	0.01	0.10		0.358	0.03	0.17		0.174	0.02	0.12		0.291

Patients’ clinical data such as neurologic status (KPS used as surrogate parameter) did not have any significant impact. Further detailed statistical analysis of the present correlations is summarized in Table 4.

## Discussion

When standard therapy options for malignant brain tumours are at the end a huge grey zone between best supportive care and another last adjuvant therapy begins. Often, patients, caregivers and the treating neurooncologists hamper to stop active treatment phase and negotiate to start another adjuvant treatment. Balancing quality of life vs. the hope to increase lifetime is maybe the most difficult situation in neurooncology. But there are only few data elucidating pros and cons of final therapy decisions. Therefore, we designed a questionnaire focusing on the evaluation of neurooncological therapy from the caregivers’ point of view after the patients have passed away. How does the caregiver retrospectively evaluate the final course of disease? Would they choose a different option if they had the chance to do it again? Would they have stopped active treatment phase earlier?

Final therapy decisions should be based on a discussion process which is led by doctors, healthcare providers, patient and caregiver. Here, we present one aspect of this process: the caregiver’s point of view. Our analysis of subjective therapy evaluation clearly underlines the significant correlation between the bond of trust between patient/caregiver/physician and the perception of the latest therapy. It also underlines the central role of caregivers, who therefore should be involved in therapy discussions.

In our patient cohort, an even number of patients died at home or in a hospice (37.8% and 38.8%, respectively). Only 23.4% spent their final days in hospital. Compared to literature, the rate of patients dying in a hospice is high. Rates of 8.5–27.9% have been reported in different European studies [6, 10, 13, 14]. The status of hospices in society has changed during the last years maybe because it has gained more acceptance. The German hospice and palliative association reports that the number of inpatient hospice and palliative care facilities has increased significantly between 1996 and 2016. In 1996, only 28 palliative care units and 30 inpatient hospices for adults were available. Until 2016 the number has increased eightfold to around 330 palliative care units and around 250 inpatient hospices. If the above cited papers are sorted by publication date, the numbers of patients who died in a hospice have constantly been rising from 8.5% in 2010 (12–19.8% in 2013) to 27.9% in 2020 [6, 10, 13, 14] which reflects the changing acceptance of hospices in society.

After transmission into the EOL phase, patient and caregivers were also transmitted to another care system, and neurooncologists are out of sight. Therefore, caregivers might feel left alone without sufficient support. Here, the majority of all caregivers (73.5%) received additional support, mainly in terms of nursing service. These data have been collected in a European country with an easily accessible, stable and well-working health service—yet it underlines that additional care is needed as well as more and more accepted by the caregivers.

Only few studies analysed the EOL from the caregiver point of view. Gonella et al. performed a recent systemic review regarding end-of-life care in nursing homes, independent from the patient’s diagnosis. Important aspects for caregivers were (a) emotional and psychosocial support, (b) to be kept informed, (c) promoting family understanding (d) and establishing a partnership with caregivers by involving and guiding them in a shared decision-making. The authors conclude that these elements improved the quality of end-of-life of both patients and caregivers, thus suggesting a common ground between good end-of-life care and palliative care [15], which is in accordance to our data.

Changing from active tumour-specific therapy to best supportive care is challenging for the neurooncologist as well as for the patients and their caregivers. A recent study reported that 25.6% were still treated tumour specifically within the last 4 weeks of life and 79.1% within the last 3 months [10]. Bahler et al. discovered that in their study cohort, almost 10% of patients started a new chemotherapy regimen in the last 4 weeks of life [8]. They discussed that palliative late-stage chemotherapy might be evaluated as an option to improve survival, especially in patients who are explicitly seeking further treatment. A recent caregiver survey of bereaved cancer patients stated that a perception of better end-of-life care was associated with earlier hospice enrollment, avoidance of ICU admissions ≤ 30 days of death and death outside the hospital [16]. In our patient cohort, 67.6% patients received oral chemotherapy as final treatment option. Although different studies report neither improvement nor negative side effects caused by adjuvant chemotherapy during the EOL phase [17], patients and caregivers seem to prefer risking additional side effects rather than completely stopping all adjuvant therapy options.

One major aspect of this survey was the overall satisfaction as an indicator for well-balanced therapy decisions. Most caregivers stated that they were satisfied with their choice of final therapy; especially in the case of surgical intervention, they would make the same decision again (85.7%). Seventy-four percent declared that they would not want any intensified therapy and that therapeutic effects predominated side effects in 57.7%. 28.7% of caregivers felt overburdened by this situation, and 8.6% would abandon any therapy during the EOL phase. Interestingly, in our analysis, caregivers comment more positive about surgery and chemotherapy compared to radiotherapy (Table 4) which might be explained by the changing health personnel. At our department, surgery and chemotherapy are performed by the same neurooncologic team, whereas for radiotherapy, patients are transferred to a different department. These evaluations correlated with the interpersonal relationship between caregivers, patients and physicians (as well as medical team), their management of anxiety and the comprehensibility of the informed consent. This significant correlation underlines the impact of medical staff. Especially in neurooncologic patients, a positive relation between medical staff, patients and their caregivers is an important factor during active therapy as well as after the transition towards the EOL phase and best supportive care. We should be aware of this significant effect and specifically teach young residence in the field of neurooncology.

## Limitations

Due to the study design (cross-sectional survey) evaluating the final treatment from the caregivers’ perspective, there are several limitations. One major problem of questionnaire survey data are the variable return rates which range mainly below 40% [6, 10, 13]. Here, the overall return rate was 47.4%. 84.4% of caregivers, who were initially contacted by phone, returned the survey data. In comparison, 26.2% of caregivers who were contacted solely by mail returned the survey. On the one hand, the importance of personal contact to optimize data return rates is underlined; on the other hand, this could bias our study population in supporting rather contend caregivers than displeased ones. In this study, the time from assessment to the patient’s death has got a huge range. Assessment was performed from 6 months to 6 years (median 2.8 years) after the patients passed away. Therefore, the caregivers’ perceptions might have changed. We analysed the subjective caregivers’ evaluation focusing on the EOL phase. Our analyses assumed that the patient would have had the same option as their caregiver and evaluated this phase and impact of treatment in the same way. Seventy-seven percent of caregivers reassured that they could reflect the patient’s perspective. The questionnaire we used was designed by a specialized team of neurosurgeons and neuro- and psycho-oncologists solely for this analysis and was not validated before.

Finally, due to the small number of caregivers, we only got a general idea of a small patient cohort. Further analyses concerning the impact of (1) the time-point from latest therapy and death and (2) the sequence of the received treatment lines were difficult because of the cohort size and need further elucidation.

## Clinical implication

Therapy of neurooncologic patients implies a complex integration of patients/caregivers and social workers by the treating physicians. Although patients and their families increasingly benefit from additional social and palliative support, the most important central point influencing the subjective therapy evaluation is a positive patient-caregiver-physician relationship. Physicians should be aware of this and integrate caregivers in future therapy decisions.

## Conclusion

Little is known about the last treatment phase of neurooncologic patients. Most studies focus on medical supportive care and dismiss the impact of active terminal tumour treatment. In this study, we analysed the final active treatment assessment from the caregivers’ perspective. Would they make the same decision again, with the knowledge and experience they now have? Our data demonstrate that (1) most caregivers positively evaluate final therapy decisions which is (2) correlated with the health personnel-patient-caregiver relationship. Therefore, this analysis from the caregiver’s perspective clearly underlines the significant correlation between patient-caregiver-physician relation and the subjective perception of the last treatment. It also illustrates the central role of caregivers during this time. Therefore, caregivers should be involved in treatment discussions in particular. Neurooncologists should be specially trained in communication techniques and psycho-oncology.

## Supplementary Information

Below is the link to the electronic supplementary material.

Supplementary file1 (PDF 184 KB)

## Data Availability

N/A.

## Code availability

N/A.

## References

[1] Stupp R, Mason WP, van den Bent MJ (2005). Radiotherapy plus concomitant and adjuvant temozolomide for glioblastoma. N Engl J Med.

[2] Herrlinger U, Tzaridis T, Mack F (2019). Lomustine-temozolomide combination therapy versus standard temozolomide therapy in patients with newly diagnosed glioblastoma with methylated MGMT promoter (CeTeG/NOA-09): a randomised, open-label, phase 3 trial. Lancet.

[3] Stupp R, Taillibert S, Kanner A (2017). Effect of tumour-treating fields plus maintenance temozolomide vs maintenance temozolomide alone on survival in patients with glioblastoma: a randomized clinical trial. JAMA.

[4] Weller M, van den Bent M, Tonn JC (2017). European Association for Neuro-Oncology (EANO) guideline on the diagnosis and treatment of adult astrocytic and oligodendroglial gliomas. Lancet Oncol.

[5] Pace A, Dirven L, Koekkoek JAF (2017). European Association for Neuro-Oncology (EANO) guidelines for palliative care in adults with glioma. Lancet Oncol.

[6] Flechl B, Ackerl M, Sax C (2013). The caregivers’ perspective on the end-of-life phase of glioblastoma patients. J Neurooncol.

[7] Voltz R, Borasio GD (1997). Palliative therapy in the terminal stage of neurological disease. J Neurol.

[8] Bahler C, Signorell A, Blozik E, Reich O (2018). Intensity of treatment in Swiss cancer patients at the end-of-life. Cancer Manag Res.

[9] Prigerson HG, Bao Y, Shah MA (2015). Chemotherapy use, performance status, and quality of life at the end of life. JAMA Oncol.

[10] Hertler C, Eisele G, Gramatzki D (2020). End-of-life care for glioma patients; the caregivers’ perspectice. J Neurooncol.

[11] Dirven L, Sizoo E, Taphoorn M (2015). Anaplastic gliomas: end-of-life care recommendations. CNS Oncol.

[12] Giammalva G, Iacopino D, Azzarello G (2018). End-of-life care in high-grade glioma patients. The Palliative and Supportive Perspective. Brain Sci.

[13] Heese O, Vogeler E, Martens T (2013). End-of-life caregivers’ perception of medical and psychological support during the final weeks of glioma patients: a questionnaire-based survey. Neuro-Oncol.

[14] Sizoo EM, Braam L, Postma TJ (2010). Symptoms and problems in the end-of-life phase of high-grade glioma patients. Neurooncology.

[15] Gonella S, Basso I, De Marinis MG, Campagna S, Giulio P (2019). Good end-of-life care in nursing home according to the family carers’ perspective: a systematic review of qualitative findings. Palliat Med.

[16] Wright A, Keating N, John Z, Ayanian J (2016). Family perspectives on aggressive cancer care near the end of life. JAMA.

[17] Prigerson HG, Bao Y, Shah MA, Paulk ME, LeBlanc TW, Schneider BJ (2015). Chemotherapy use, performance status, and quality of life at the end of life. JAMA Oncol.

