# Supplementary material for: Would they do it again? Final treatment decisions in malignant brain tumour patients—a caregiver’s perspective
Source: Support Care Cancer. 2022 Jan 21;30(5):3985–93. doi: 10.1007/s00520-022-06796-y (PMC8942917; doi:10.1007/s00520-022-06796-y)
Supplement: Supplementary file 1 — Supplementary file1 (PDF 184 KB) [file 520_2022_6796_MOESM1_ESM.pdf]

Questionnaire

on the study: "The therapy of malignant brain tumors from the perspective of the relatives- Questionnaire assessment to experience the last days / weeks "

Patient-ID: _______________

Relationship to the deceased: ☐ Parents ☐Siblings ☐Child ☐Life partner

Nationality: _______________

Age of the deceased: ____________________

In the following, please state your satisfaction in the above-mentioned subject areas during the treatment of your relative.

How do you rate in retrospect (1 fully satisfied, 5 not at all satisfied)

- **Interpersonal dealings with your loved one**

→ on the part of the doctors ☐ 1 ☐ 2 ☐ 3 ☐ 4 ☐ 5

→ on the part of the nurses ☐ 1 ☐ 2 ☐ 3 ☐ 4 ☐ 5

- **Informed consent**

→ comprehensibility ☐ 1 ☐ 2 ☐ 3 ☐ 4 ☐ 5

→ time point ☐ 1 ☐ 2 ☐ 3 ☐ 4 ☐ 5

→ overall impression ☐ 1 ☐ 2 ☐ 3 ☐ 4 ☐ 5

→ dealing with questions ☐ 1 ☐ 2 ☐ 3 ☐ 4 ☐ 5

- **Contact between doctors or the hospital and the patient**

☐ 1 ☐ 2 ☐ 3 ☐ 4 ☐ 5

- **Informed consent about any interventions /therapies**

→ Surgery	☐ 1	☐ 2	☐ 3	☐ 4	☐ 5
→ Radiotherapy	☐ 1	☐ 2	☐ 3	☐ 4	☐ 5
→ Chemotherapy	☐ 1	☐ 2	☐ 3	☐ 4	☐ 5

- **Dealing with fears**

→ of the patients	☐ 1	☐ 2	☐ 3	☐ 4	☐ 5
→ your own	☐ 1	☐ 2	☐ 3	☐ 4	☐ 5

- **Connection to other facilities**

→ Rehabilitation	☐ 1	☐ 2	☐ 3	☐ 4	☐ 5
→ Palliative Care	☐ 1	☐ 2	☐ 3	☐ 4	☐ 5
→ Hospice	☐ 1	☐ 2	☐ 3	☐ 4	☐ 5

Where did your relative died?

- Hospital ☐ Department of neurosurgery ☐ other wards
- Hospice ☐
- Palliative Care ☐
- At home ☐

If your relative died at home:

→ What support did you get?

☐Home help ☐Nursing ☐Driving service ☐psychological support

→ How was this support brought to you?

☐from medical side ☐from nursing side ☐other

→ Were you satisfied with the support?

☐yes ☐no

→ Would you have wished for further support?

☐yes ☐no

→ if yes, which?: ____

→ Did your loved one came home after the last neurosurgical intervention before he passed away?

☐yes ☐no

If your relative died in a hospice:

→ What support did you get?

☐Home help ☐Nursing ☐Driving service ☐psychological support

→ How was this support brought to you?

☐from medical side ☐from nursing side ☐other

→ Would you have wished for further support from the neurosurgical team?

☐yes ☐no

→ If yes, what should this support have looked like?

____

If your relative died at palliative care:

→ Which support did you get?

☐Home help ☐Nursing ☐Driving service ☐psychological support

→ How was this support brought to you?

☐from medical side ☐from nursing side ☐other

→ Would you have wished for further support?

☐yes ☐no

If your relative died at the department of neurosurgery:

→ Which support did you get?

☐Home help ☐Nursing ☐Driving service ☐psychological support

→ How was this support brought to you?

☐from medical side ☐from nursing side ☐other

→ Would you have wished for further support?

☐yes ☐no

Looking back, how do you rate the LAST therapy measures taken??
(1 fully satisfied, 5 not at all satisfied)

How satisfied were you with

	1	2	3	4	5
Surgery:	☐	☐	☐	☐	☐

Chemotherapy: ☐ 1 ☐ 2 ☐ 3 ☐ 4 ☐ 5

Radiation: ☐ 1 ☐ 2 ☐ 3 ☐ 4 ☐ 5

Would you choose the same therapy again?

Surgery: ☐yes ☐ no, because ____

Chemotherapy: ☐yes ☐ no, because ____

Radiation: ☐yes ☐no, because____

In your opinion, did the therapeutic effect outweigh the side effects?

☐yes ☐ no, because ____

Have you given yourself false hopes through the proposed therapeutic measures?

☐yes ☐no

Looking back, would you say that in the last few weeks of his or her life your loved one would have wished for a different decision about the therapeutic measures that did not correspond to your own attitude?

☐yes ☐no

All in all, were you overwhelmed with the course of therapy?

☐yes ☐no

All in all, would you have wished for a different therapy?

☐yes ☐no

In retrospect, which therapies would you forego?

☐Surgery ☐Chemotherapy ☐Radiation

In retrospect, which therapy would you prefer?

☐Surgery ☐Chemotherapy ☐Radiation

Remarks:

____

____

____

____
